# Birth Weight, School Sports Ability, and Adulthood Leisure-Time Physical Activity

**DOI:** 10.1249/MSS.0000000000001077

**Published:** 2017-01

**Authors:** Ahmed Elhakeem, Rachel Cooper, David Bann, Diana Kuh, Rebecca Hardy

**Affiliations:** 1 MRC Unit for Lifelong Health and Ageing at UCL, London, UNITED KINGDOM; 2 Centre for Longitudinal Studies, UCL Institute of Education, London, UNITED KINGDOM

**Keywords:** Birth Weight, Life Course, Exercise, Sports, Physical Activity

## Abstract

**Purpose:**

This study aimed to examine the associations of birth weight with ability in school sports in adolescence and participation in leisure-time physical activity (LTPA) across adulthood and to investigate whether associations between birth weight and LTPA change with age.

**Methods:**

Study participants were British singletons born in 1946 and followed up to age 68 yr (the Medical Research Council National Survey of Health and Development). Birth weights were extracted from birth records. Teacher reports of ability in school sports were collected at age 13 yr. LTPA was self-reported at ages 36, 43, 53, 60–64, and 68 yr and categorized at each age as participating in sports, exercise, and other vigorous LTPA at least once per month versus no participation. Associations were examined using standard and mixed-effects logistic regression models.

**Results:**

Relevant data were available for 2739 study participants (50.1% female). When compared with the low birth weight group (≤2.50 kg), those with heavier birth weights were more likely to be rated as above average or average at school sports (vs below average); fully adjusted odds ratio = 1.78 (95% confidence interval = 1.14–2.77). Across adulthood, those with heavier birth weights were more likely to participate in LTPA than those with low birth weight; fully adjusted odds ratio of LTPA across adulthood = 1.52 (95% confidence interval = 1.09–2.14). This association did not vary by age (*P* = 0.5 for birth weight by age interaction).

**Conclusions:**

Low birth weight was associated with lower ability in school sports and with nonparticipation in LTPA across adulthood. Identifying the underlying developmental and social processes operating across life for low birth weight infants may inform the design of appropriate interventions to support participation in LTPA across life.

Regular leisure-time physical activity (LTPA) provides many health benefits that include reduced rates of early death from chronic disease, whereas physical inactivity is a major contributor to morbidity and premature mortality (2,39). To develop interventions that are more effective at promoting LTPA, a better understanding of its determinants from across life is required (6,10,13,19). Studies taking a life course approach suggest that biological and social factors in early life may influence adult health-related behaviors, including LTPA (3,6,7,10,13,19,20,29,30). These include studies that have examined hypothesized associations between size at birth (as a marker of exposures *in utero*) and physical activity across different phases of life (1,3,18,26,28,30,31,33).

The fetal origins of adult disease hypothesis (5) suggests that *in utero* experiences such as undernutrition can, in addition to limiting size at birth, alter development and increase susceptibility to chronic disease. Lower birth weight has been associated with higher risks of cardiovascular disease (5,38) and type II diabetes (41), and more recently also with lower aerobic fitness and muscular endurance (32) and less favorable body composition in terms of lower bone (23) and muscle mass (4), and weaker muscle strength (12). Low birth weight has also been associated with poorer motor and cognitive development (27).

These findings (4,5,12,23,27,32,38,41) alongside animal studies showing less physical activity in those born to undernourished mothers (42) provide evidence to suggest that adolescents and adults with low birth weight might participate less in LTPA. The underlying mechanisms may operate through an effect on the motor skills required to develop competence at sports, a reduced exercise capacity and subsequent self-selection out of sports and exercise in those born with low birth weight (11,13). Moreover, an impaired exercise capacity is also a reported consequence of reduced gestational age (i.e., preterm birth) (11,13), and thus any associations between low birth weight and less LTPA could be driven by an intrauterine growth restriction, a reduced gestational age, or a combination of both.

Of the existing epidemiological studies that have investigated associations between birth weight and physical activity, inconsistent results are reported with a tendency to find null associations (26,28,29,31) or less LTPA in those born with low birth weight (1,3,18,33). Most studies have examined activity in childhood, adolescence, or young adulthood and/or rely on a single measure of physical activity. However, the influence of birth weight on chronic disease risk is more apparent later in life (38,41), so it could be that associations with LTPA might also be more apparent in adulthood. Thus, studies that extend into and across adulthood are required. In addition, the assessment of whether any associations found change across adult life may help establish underlying mechanisms that could have important implications for future intervention. Therefore, the aim of this study was to examine the associations of birth weight with ability in school sports in adolescence and participation in LTPA across adulthood and to investigate whether the association between birth weight and LTPA changes with age.

## Methods

### Study Population

Study participants were from the Medical Research Council (MRC) National Survey of Health and Development (NSHD), a nationally representative sample of 5362 British singleton births during 1 wk in March 1946 and followed up regularly across childhood and adulthood (21). At age 13 yr (1959), the school teacher who was most familiar with each study participant completed a school-based questionnaire. At ages 36 yr (1982), 43 yr (1989), and 53 yr (1999), trained nurses interviewed and assessed the study participants in their own homes. At age 60–64 yr (2006– 2010), they attended one of six clinical research facilities or received a home visit, and at age 68 yr (2014), they completed a postal questionnaire.

Of those successfully contacted at ages 36 yr (*n* = 3322), 43 yr (*n* = 3262), 53 yr (*n* = 3035), 60–64 yr (*n* = 2661), and 68 yr (*n* = 2453), 99.6%, 100%, 98.4%, 82.2%, and 99.1%, respectively, provided information on LTPA (3766 participants had at least one measure of LTPA). The participating samples at ages 53 and 60–64 yr have been found to be broadly representative of similar age members of the general UK population (34,37). At the last completed round of data collection at age 68 yr, 83.4% of 2943 study participants who were still alive and eligible to participate were successfully contacted. Of the 490 who were not successfully contacted, 11 had died, 453 did not return a questionnaire, and 26 questionnaires were returned undelivered.

Relevant ethics approval has been granted for each data collection; ethical approval for the most recent assessment in 2014 was obtained from the Queen Square Research Ethics Committee (14/LO/1073) and the Scotland A Research Ethics Committee (14/SS/1009). Study participants provided written informed consent.

### Measurements

#### Birth weight

Birth weight, recorded to the nearest quarter of a pound, was extracted from birth records within 6 wk of delivery and subsequently converted to kg.

#### School sports ability and adulthood LTPA

When study participants were 13 yr old, teachers were asked to complete a questionnaire rating their ability in school sports as above average, average, or below average compared with their peers (20). This measure is used as a marker of study participants’ overall ability at school-based games and activities requiring competence in motor skills and coordination (e.g., team sports, physical education, and athletics) and was previously shown to relate to LTPA at age 36 yr (20).

At ages 36, 43, 53, 60–64, and 68 yr, study participants reported how often they participated in LTPA during nurse interviews or using self-completed questionnaires. At age 36 yr, study participants reported the number of times they took part in 27 different sports, exercises, and other leisure activities during the previous month using questions based on the Minnesota LTPA questionnaire (20). At age 43 yr, information was collected on participation in sports, exercise, or other vigorous leisure activities in the previous year including for how many months and how often in those months activities were performed (9). At age 53 yr, study participants were asked how often they participated in sports, exercise, or other vigorous leisure activities during the previous 4 wk, and this question was asked again at ages 60–64 and 68 yr. At each age, study participants were classed as inactive if they reported no participation in LTPA, moderately active if they participated up to four times in LTPA, or regularly active if they reported taking part five or more times in LTPA (in the previous month at age 36 yr, per month at age 43 yr, and in the previous 4 wk at ages 53, 60–64, and 68 yr).

#### Covariates

Birth order, childhood socioeconomic position, and cognitive ability were identified as potential confounders based on existing literature (6,14,24). On the basis of mother’s report of birth order, study participants were classified as first, second, or third or later born. Father’s Registrar General’s occupational class at age 4 yr was used to indicate socioeconomic position in childhood and was grouped into four categories (I and II: professional, managerial, or technical; IIINM: skilled nonmanual; IIIM: skilled manual; and IV and V: partly skilled or unskilled). Cognitive ability was tested at age 8 yr in a school setting and included vocabulary, comprehension, and reading tests from which a standardized cognitive score was derived. Missing paternal occupational class (*n* = 173) and cognition scores (*n* = 199) were imputed with values recorded at ages 11 and 15 yr.

In addition to the previously mentioned covariates, sports ability in childhood and physical health in adulthood were hypothesized to mediate associations between birth weight and adulthood LTPA. Physical health was derived from information collected during nurse interviews at age 36 yr on weight, disability, self-reported health problems, hypertension, lung function, and incidence of hospital admissions (22). This information was used to categorize participants into worst, intermediate, or best physical health (22). Supplementary analyses in those with information on body mass index (BMI) were also conducted to examine whether body size mediated associations. BMI (kg·m^−2^) was derived from height and weight measured using standardized protocols in childhood at age 11 yr and in adulthood at age 36 yr.

### Statistical Analysis

Formal tests of interactions between sex and birth weight were undertaken, and subsequent analyses were adjusted for sex after no evidence of interaction was found. Formal tests of deviation from linearity were performed and showed evidence of nonlinear associations between birth weight and sports ability and LTPA. As a result, study participants were grouped into five categories of birth weight (≤2.50 kg, 2.51–3.00 kg, 3.01–3.50 kg, 3.51–4.00 kg, and > 4.00 kg), and the low birth weight group (≤2.50 kg) was used as the reference in analyses. For these analyses, dichotomous measures of sports ability and LTPA were derived. The two groups of above average and average ability in school sports were combined and compared with the group with below average ability. At each age in adulthood, the two groups reporting moderate and regular participation in LTPA were combined and compared with the group that reported no participation in LTPA.

We examined how birth weight relates to ability in school sports using logistic regression. The association between birth weight and LTPA across adulthood was examined using mixed-effects binary logistic regression with random intercepts and slopes for age so that we could include all participants with at least one measure of LTPA while also accounting for correlations among repeated LTPA responses (25). In all models, we included sex and a sex-by-age interaction. To investigate whether the association between birth weight and probability of being active in LTPA changes with increasing age, we added a birth weight by age interaction term to the mixed-effects models. The estimated fixed-effects coefficients were used to plot the log-odds of LTPA for each birth weight group against age. In addition, multinomial mixed-effects models (25) estimated using a simulation approach were fit to the categorical LTPA data to examine the associations of birth weight with moderate and regular participation in LTPA (see Table, Supplementary Digital Content 1, Associations between birth weight and moderate and regular participation in LTPA, http://links.lww.com/MSS/A745).

Models of the associations of birth weight with both sports ability and LTPA were first adjusted for sex and then for birth order, socioeconomic position, and cognitive ability. An additional model was then run when examining the association between birth weight and LTPA, which included adjustment for ability in school sports and physical health in adulthood. Lastly, we reran both final models in those with data on BMI and compared estimates to models with added adjustment for BMI. Sports ability models were adjusted for childhood BMI, whereas LTPA models were adjusted for adult BMI. Analyses were conducted in STATA 14 (StataCorp, College Station, TX).

## Results

### Characteristics of study participants

A total of 2739 study participants had at least one measure of adulthood LTPA and complete data on ability in school sports, birth weight, and all selected covariates (Table 1). The majority of included participants had LTPA data from four of the five different ages in adulthood (a total of 10,980 LTPA assessments between ages 36 and 68 yr were included in analyses). When compared with those excluded because of missing LTPA data (*n* = 1596), higher proportions of those with at least one measure of LTPA were female (49.6% vs 42.5%) and had fathers in occupational classes I and II (23.1% vs 20.9%), and lower proportions had low birth weight (4.7% vs 8.8%). At age 13 yr, higher proportions of girls were rated as above average or average in school sports (Table 1). At ages 36 and 43 yr, higher proportions of men reported taking part in LTPA, but sex differences were less marked at older ages (Table 1).

### Birth weight and ability in school sports at age 13 yr

When compared with the low birth weight group (≤2.50 kg), those in other heavier birth weight categories were more likely to be rated as above average or average (vs below average) at school sports at age 13 yr (Table 2). For example, the sex-adjusted odds ratio (OR) of above average or average sports ability was 1.89 (95% confidence interval [CI] = 1.22–2.93) when combining the four other birth weight groups and comparing this larger group with the low birth weight group. This association was only slightly attenuated when models were adjusted for birth order, cognitive ability, and socioeconomic position (Table 2) (fully adjusted OR of above average or average sports ability = 1.78; 95% CI = 1.14–2.77, when comparing those in all heavier birth weight groups with the low birth weight group). Further adjustment for childhood BMI slightly strengthened this association (see Table S2A, Supplementary Digital Content 2, Associations between birth weight and ability in school sports at 13 yr and participation in LTPA, http://links.lww.com/MSS/A746).

### Birth weight and LTPA between ages 36 and 68 yr

**Figure 1:**
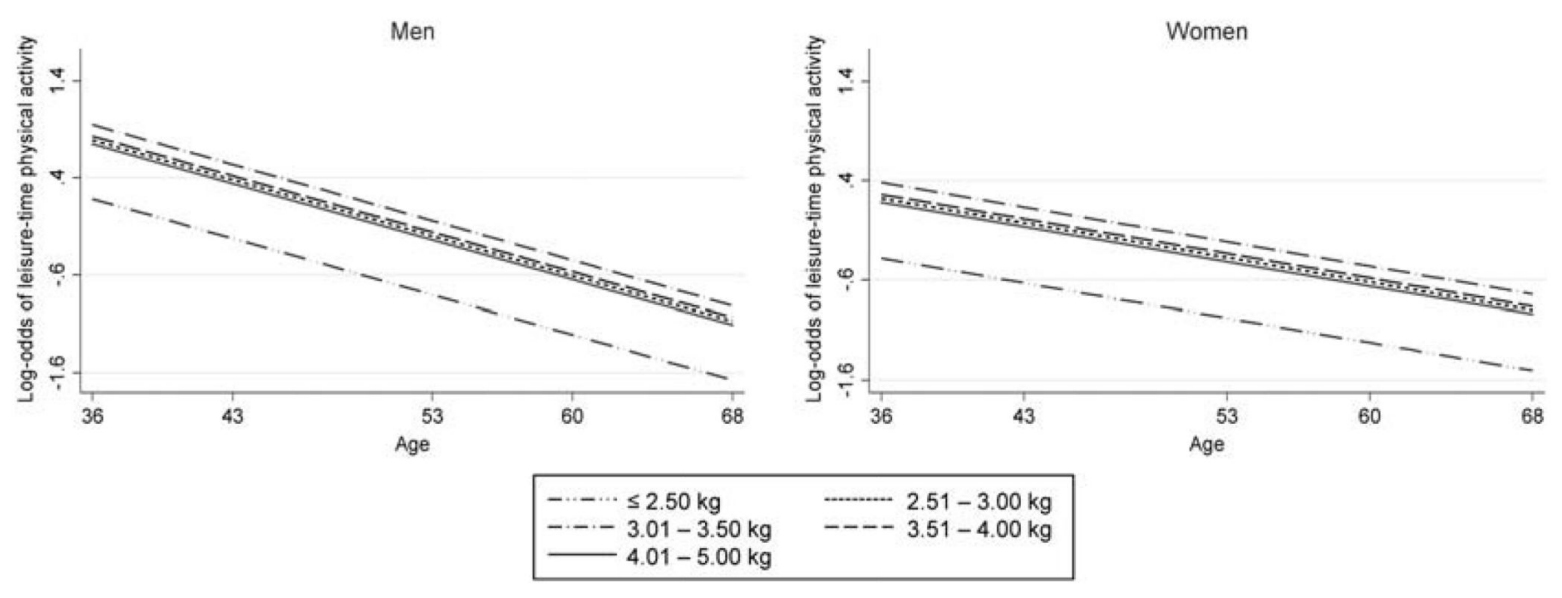
Log-odds of LTPA for each birth weight group by age in the MRC NSHD. Plots are presented separately for men and women because of a sex–age interaction, which means that the decline in LTPA is greater in men than women.

When compared with the low birth weight group, those in all heavier birth weight groups were more likely to participate in LTPA across adulthood (Table 2). There was no evidence of an interaction between birth weight and age (*P* = 0.5 for continuous and categorical birth weight by age interactions), suggesting these associations did not differ by age at assessment of LTPA. This is consistent with the finding of similar OR of LTPA at each age in adulthood in study participants with nonmissing LTPA (see Table, Supplementary Digital Content 3, Associations between birth weight and LTPA at each adult age in study participants, http://links.lww.com/MSS/A747). A plot of the log-odds of LTPA shows the decline in likelihood of activity with age for all five birth weight groups, with the lowest birth weight group always having lower probability of LTPA (Fig. 1). This plot also shows that although men were more likely to report LTPA earlier in adulthood, women had less pronounced decline in LTPA across adulthood in all birth weight groups (Fig. 1).

The association between birth weight and LTPA was slightly attenuated by adjustment for early life covariates and to a lesser degree by further adjustment for ability in school sports and physical health in adulthood (Table 2). The sex-adjusted OR of adulthood LTPA was 1.92 (95% CI = 1.35–2.73) when comparing those in the group combining the four heavier birth weight groups with the low birth weight group, which was attenuated to 1.63 (95% CI = 1.16–2.29) after adjustment for early life covariates, and to 1.52 (95% CI = 1.09–2.14) in the fully adjusted model. Associations between low birth weight and lower likelihood of participation in LTPA were stronger with more frequent participation (see Table, Supplementary Digital Content 1, Associations between birth weight and moderate and regular participation in LTPA, http://links.lww.com/MSS/A745). The sex-adjusted relative risk ratios (95% CI) of moderate (1–4 times per month) and regular (5 or more times per month) LTPA across adulthood (vs no participation) were 1.60 (1.24–2.12) and 2.83 (1.87–4.37), respectively, when comparing all four heavier birth weight groups with the low birth weight group. These estimates were attenuated to 1.25 (0.90–1.93) and 1.81 (1.23–2.57), respectively, after full adjustment. Additional adjustment for adulthood BMI had little influence on findings (see Table S2B, Supplementary Digital Content 2, Associations between birth weight and ability in school sports at 13 yr and participation in LTPA, http://links.lww.com/MSS/A746).

## Discussion

We examined prospectively collected data from the longest running British birth cohort and found that low birth weight was associated with lower ability in school sports and with nonparticipation in LTPA across adulthood. More precisely, our findings showed that, when compared with low birth weight, those in heavier birth weight groups were more likely to be rated by their school teacher as above average or average rather than below average in sports at age 13 yr and were more likely to participate in LTPA between ages 36 and 68 yr. These associations were only partly attenuated after adjustment for a range of covariates.

### Comparison with other studies

Some of the other studies that have previously investigated this association in younger more recently born cohorts support our findings (1,3,18). These include a meta-analysis which showed that Scandinavian adolescents and adults in lower birth weight groups (range = 1.26–2.75 kg) were less likely to participate in LTPA than the reference birth weight group (3.26–3.75 kg) (1) and higher levels of leisure-time physical inactivity reported by 23-yr-old Brazilian women born in 1982 with low birth weight (<2.50 kg) (3). Also supporting our results are findings of less participation in outdoor sporting activity by 12-yr-old Australian adolescents with low birth weight (<2.00 kg) that persisted during 5-yr follow-up (18).

We found that associations between low birth weight and lower likelihood of LTPA were consistent across adult life, which is similar to animal studies showing offspring from undernourished mothers to be less physically active across life, including at older adult ages, when compared with normal offspring (42). That associations between birth weight and LTPA were apparent at older ages is similar to findings from a Finnish study showing older adults with an average age of 62 yr reported higher intensity LTPA if they were bigger at birth in terms of weight and length (33).

The results presented here are not consistent with null associations reported between birth weight and physical activity levels in children (26,28). However, as associations between birth weight and chronic disease tend to be more apparent later in life (38,41); this may also be the case for LTPA (1,3,18,33). Likewise, it is also thought that associations between preterm birth and LTPA tend to be more apparent in adulthood and adolescence than in childhood (11). Our findings are also in contrast to a study from the next oldest British birth cohort born in 1958 where the authors reported that no differences were found between low (<2.50 kg) and heavier birth weights in levels of LTPA assessed in middle adulthood, but no estimate was provided (30).

### Explanation of findings

Our findings suggest that prenatal growth may influence sports ability and participation in LTPA across life (8,13). Motor deficits including difficulties in movement-related tasks and other neurocognitive impairments have been reported in children with low birth weight (27), which may explain the associations found here with lower ability in school sports. A lower sports ability in early life has also been shown to be associated with less LTPA in adulthood (20,29,30); however, adjustment for sports ability only marginally reduced the association between birth weight and adulthood LTPA. Thus, other pathways besides tracking of physical activity into adulthood are likely involved in explaining that finding.

Poorer levels of motor skills and coordination (27), less favorable body composition (4,23), including weaker muscle strength (12), and more prevalent chronic disease (5,38,41) in those with low birth weight may contribute to their higher probability of nonparticipation in LTPA across adulthood. Yet the associations found in this study between birth weight and LTPA were consistent across adulthood, which suggests that health conditions related to birth weight may not play a major role in explaining these findings. Consistent with this, adjusting for adult physical health problems only slightly weakened the association between birth weight and LTPA.

### Methodological considerations

There was some loss to follow-up in NSHD, as expected in long-running studies. This led to only slight differences in characteristics between those included and those with missing data. A survival selection bias where those healthiest and physically active from the low birth weight group survive to an older age is possible and may have biased results toward the null. However, our modeling strategy maximizes sample size and improves precision of estimates of association as all individuals with at least one measure of LTPA are included under the missing at random assumption (25). Other important strengths of this study included the collection of measured birth weights from birth records within weeks of delivery, an adjustment for important and prospectively ascertained covariates, and an investigation of age-related changes in associations with LTPA. In addition, we were also able to examine the relationship between birth weights and an indicator of sports ability from early life and to compare findings with those of participation in LTPA across adulthood.

One limitation of this study is that information on gestational age was not available, and therefore we could not distinguish between those born small-for-gestational age and those with low birth weight due to preterm birth. However, there would have been less variation in gestational age in this study population than that in more recent born cohorts as preterm births were less likely to survive in the 1940s (36). As a result of reduced survival, there were a limited number of participants classified as having low birth weight in our study, but despite reduced statistical power, associations were observed with both outcomes. In addition, birth weight is the only measure of birth size available in NSHD and is only a proxy marker of the adaptations that a fetus may make to its body’s structure and function in response to stress experienced *in utero*.

Self-reported LTPA can be subject to recall and misclassification errors; therefore, a cautious interpretation of the findings is necessary. However, questionnaires/interviews help capture contextual circumstances surrounding physical activity, making them suitable for widespread gathering of data on activity types and domains such as LTPA (35). If there was differential reporting of LTPA by birth weight groups, this could bias our findings; however, we have no reason to suspect this to be the case. Furthermore, when self-reported physical activity and data from activity monitors were compared in a subsample of this cohort, they were found to rank study participants similarly by levels of physical activity (15). Those participating in LTPA across adulthood were also found to spend greater amounts of time in moderate-to-vigorous intensity activity as assessed by these monitors when compared with others reporting no LTPA (17). The LTPA measures used here were relatively crude in that they summarized activity according to whether any participation in LTPA was reported. However, even small amounts of LTPA are important for health particularly in older populations (2,39). Further, in supplementary analyses, we were able to show a dose–response nature to this association, suggesting those with low birth weights were even less likely to report higher levels of LTPA across adulthood.

### Implications

It is important to recognize that those with low birth weight may require more support than others if they are to achieve sufficient physical activity across life to realize its health benefits (2,16,39). The associations observed are likely to be generalizable to more recently born cohorts because associations have been seen in the same direction in younger cohorts (1,3,18). The increasing long-term survival rates of babies born with low birth weight (36,40) means that there are increasing numbers of adults who were born with low birth weight and thus there may be a growing proportion of the population who are at greater risk of having lower competence at sports and who are unlikely to be participating in LTPA. Designing appropriate interventions may require a better understanding of how related processes such as postnatal growth, motor capability, and body composition influence physical activity in those with low birth weight. Furthermore, to allow more meaningful comparison of findings between different cohorts, it would be useful to harmonize methods of analyses.

In conclusion, findings from this 68-yr prospective follow-up study of the 1946 British birth cohort showed that, when compared with those with low birth weight, other study participants with heavier birth weights were less likely to be rated by their school teacher as below average at sports and were more likely to participate in LTPA across adulthood. Understanding the underlying biological, developmental, and social processes that explain the relationship between low birth weight and low sports ability and nonparticipation in LTPA may help identify appropriate characteristics of effective interventions.

## Supplementary Material

Supplemental digital content is available for this article. Direct URL citations appear in the printed text and are provided in the HTML and PDF versions of this article on the journal’s Web site (www.acsm-msse.org).

Supplementary Digital Content 1

Supplementary Digital Content 2

Supplementary Digital Content 3

## Figures and Tables

**Table 1:** Characteristics of study participants with relevant data from the MRC NSHD overall and by sex (*n* = 2739).

	Overall, *N* (%)	Males, *n* (%)	Females, *n* (%)	*P* value of Sex Difference Test
Birth weight (kg)				*P* < 0.001
≤2.50	120 (4.4)	42 (3.1)	78 (5.7)	
2.51–3.00	455 (16.6)	180 (13.2)	275 (20.0)	
3.01–3.50	974 (35.6)	466 (34.1)	508 (37.0)	
3.51–4.00	906 (33.1)	488 (35.7)	418 (30.5)	
>4.00	284 (10.4)	191 (14.0)	93 (6.8)	
Ability in school sports age 13 yr				*P* = 0.001
Above average or average ability	2327 (85.0)	1130 (82.7)	1197 (87.2)	
Below average ability	412 (15.0)	237 (17.3)	175 (12.8)	
LTPA across adulthood^a^					
Age 36 yr	1725 (63.0)	941 (68.9)	784 (57.2)	*P* < 0.001
Age 43 yr	1165 (47.0)	635 (51.2)	530 (42.9)	*P* < 0.001
Age 53 yr	1134 (50.5)	568 (52.0)	566 (49.1)	*P* = 0.2
Age 60–64 yr	606 (35.6)	277 (33.7)	329 (37.4)	*P* = 0.1
Age 68 yr	725 (39.9)	345 (39.9)	380 (39.8)	*P* = 0.9
Birth order				*P* = 0.7
First born	1129 (41.2)	569 (41.6)	560 (40.8)	
Second born	905 (33.0)	441 (32.3)	464 (33.8)	
Third or later born	705 (25.7)	357 (26.1)	348 (25.4)	
Father’s occupational class age 4 yr				*P* = 0.9
Professional/managerial/technical	601 (22.0)	300 (22.0)	301 (22.0)	
Skilled nonmanual	503 (18.4)	246 (18.0)	257 (18.7)	
Skilled manual	842 (30.7)	416 (30.4)	426 (31.1)	
Partly skilled or unskilled	793 (29.0)	405 (29.6)	388 (28.3)	
Cognitive ability age 8 yr^b^	2739 (100)	−0.01 (0.8)	0.04 (0.8)	*P* = 0.1
Physical health age 36 yr				*P* = 0.7
Worst	710 (25.9)	356 (26.0)	354 (25.8)	
Intermediate	1748 (63.8)	864 (63.2)	884 (64.4)	
Best	281 (10.3)	147 (10.8)	134 (9.8)	

≤2.50 kg: range = 1.25–2.50 kg (mean = 2.30 kg); >4.00 kg: range = 4.09–5.00 kg (mean = 4.32 kg).

a Proportions are for those taking part (once or more than once per month) in LTPA at each age.

b Data show mean (SD) standardized cognitive ability *z*-score (overall mean = 0; overall SD = 1).

**Table 2:** Associations between birth weight and ability in school sports at 13 yr and participation in LTPA between 36 and 68 yr in the MRC NSHD (*n* = 2739).

	OR (95% CI) of Above Average or Average Ability in Sports vs Below Average Ability		OR (95% CI) of LTPA (at Least Once per Month) Across Adulthood vs No LTPA		
	Model 1	Model 2	Model 1	Model 2	Model 3
Birth weight group (kg)					
≤2.50	1.00 (reference)	1.00 (reference)	1.00 (reference)	1.00 (reference)	1.00 (reference)
2.51–3.00	1.97 (1.19–3.25)	1.91 (1.15–3.16)	1.74 (1.18–2.56)	1.50 (1.03–2.17)	1.40 (0.97–2.03)
3.01–3.50	2.01 (1.26–3.19)	1.91 (1.20–3.05)	2.14 (1.49–3.08)	1.82 (1.28–2.59)	1.69 (1.19–2.39)
3.51–4.00	1.82 (1.14–2.89)	1.66 (1.04–2.66)	1.84 (1.27–2.65)	1.52 (1.06–2.16)	1.43 (1.01–2.03)
>4.00	1.60 (0.95–2.72)	1.41 (0.82–2.42)	1.74 (1.15–2.63)	1.54 (1.03–2.30)	1.47 (0.98–2.19)

Model 1: adjusted for sex. Model 2: adjusted for sex, birth order, cognitive ability, and father’s occupational class. Model 3: same as model 2 plus additional adjustment for ability in school sports and physical health in adulthood. ≤2.50 kg: range = 1.25–2.50 kg (mean = 2.30 kg); >4.00 kg: range = 4.09–5.00 kg (mean = 4.32 kg).
