# Supplementary material for: Birth Weight, School Sports Ability, and Adulthood Leisure-Time Physical Activity
Source: Med Sci Sports Exerc. Author manuscript; Available in PMC 2017 Jan 3. (PMC5207304; doi:10.1249/MSS.0000000000001077)
Supplement: Supplementary Digital Content 1 [file NIHMS69595-supplement-Supplementary_Digital_Content_1.pdf]

Supplementary Digital Content 1 Associations between birth weight and moderate and regular participation in leisure-time physical activity (LTPA) between ages 36–68 years in the MRC National Survey of Health and Development (n=2,739).

	RRR (95% CrI) of moderate LTPA (1-4 times per month) versus no LTPA			RRR (95% CrI) of regular LTPA (5 or more times per month) versus no LTPA		
	Model 1	Model 2	Model 3	Model 1	Model 2	Model 3
Birth weight (kg)						
≤ 2.50	1.00 (reference)	1.00 (reference)	1.00 (reference)	1.00 (reference)	1.00 (reference)	1.00 (reference)
2.51-3.00	1.50 (1.10 – 2.05)	1.31 (0.91 – 1.83)	1.23 (0.86 – 1.75)	2.27 (1.40 – 3.56)	1.85 (1.29 – 2.77)	1.78 (1.16 – 2.55)
3.01-3.50	1.69 (1.29 – 2.23)	1.48 (1.10 – 2.12)	1.35 (1.00 – 1.98)	2.78 (1.91 – 4.00)	2.28 (1.62 – 3.40)	2.06 (1.48 – 2.81)
3.51-4.00	1.45 (1.11 – 1.94)	1.23 (0.92 – 1.73)	1.15 (0.82 – 1.64)	2.57 (1.70 – 3.78)	2.02 (1.41 – 2.80)	1.89 (1.30 – 2.69)
> 4.00	1.34 (0.95 – 1.86)	1.24 (0.84 – 1.76)	1.16 (0.79 – 1.68)	2.35 (1.50 – 3.60)	2.04 (1.33 – 2.99)	1.92 (1.23 – 2.78)

RRR: Relative risk ratio. 95% CrI: 95% Bayesian credible intervals. Model 1: adjusted for sex. Model 2: adjusted for sex, birth order, cognitive ability and father's occupational class. Model 3: as for model 2 plus additional adjustment for ability in school sports and physical health in adulthood. See next page for methods.

Methods

The relative risk ratios of moderate (1-4 times per month) and regular (5 or more times per month) participation in LTPA with those reporting no LTPA as reference group were calculated using mixed-effects multinomial logistic regression models with individually varying intercepts (1, 2). These models were estimated with Markov Chain Monte Carlo (MCMC) simulation (3-5) as implemented in the MLwiN software v2.36 (Centre for Multilevel Modelling, University of Bristol) (4) and analyses were carried out from within STATA using the runmlwin command (5). Parameter estimates from iterative generalised least squares and marginal quasilikelihood estimation models were specified as initial values for the MCMC estimation models. Diffuse (uninformative) prior distributions were used to approximate maximum likelihood estimation and 500 iterations for the burn-in period and 5000 iterations for the monitoring period were specified (4, 5).

References

1. Liu X. *Methods and Applications of Longitudinal Data Analysis*. Academic Press: Elsevier; 2015. p. 343-77.
2. Hedeker D. A mixed-effects multinomial logistic regression model. *Stat Med* 2003;22(9):1433-46.
3. Hamra G, MacLehose R, Richardson D. Markov chain Monte Carlo: an introduction for epidemiologists. *Int J Epidemiol* 2013;42(2):627-34.
4. Browne, W.J. MCMC Estimation in MLwiN, v2.36. Centre for Multilevel Modelling, University of Bristol. 2016. Available at: http://www.bristol.ac.uk/cmm/media/software/mlwin/downloads/manuals/2-36/mcmc-web.pdf.
5. Leckie G, Charlton C. runmlwin - A program to run the MLwiN multilevel modelling software from within Stata. *J Stat Softw* 2013;52(11):1-40.
