# Supplementary material for: Birth Weight, School Sports Ability, and Adulthood Leisure-Time Physical Activity
Source: Med Sci Sports Exerc. Author manuscript; Available in PMC 2017 Jan 3. (PMC5207304; doi:10.1249/MSS.0000000000001077)
Supplement: Supplementary Digital Content 2 [file NIHMS69595-supplement-Supplementary_Digital_Content_2.pdf]

Supplementary Digital Content 2 Associations between birth weight and ability in school sports at 13 years and participation in leisure-time physical activity between 36–68 years after additional adjustment for body mass index (BMI).

Table S2A Birth weight and ability in school sports at 13 years

	OR (95% CI) of above average or average ability in sports versus below average ability	
	Model A	Model B
Birth weight group (kg)		
≤ 2.50 (n=180)	1.00 (reference)	1.00 (reference)
2.51-3.00 (n=657)	1.59 (1.04 – 2.42)	1.63 (1.07 – 2.49)
3.01-3.50 (n=1355)	1.44 (0.98 – 2.13)	1.52 (1.03 – 2.26)
3.51-4.00 (n=1288)	1.51 (1.01 – 2.24)	1.64 (1.10 – 2.44)
> 4.00 (n=431)	1.33 (0.85 – 2.09)	1.50 (0.95 – 2.35)

N=3911 (sample comprises those with data on birth weight, ability in school sports, birth order, cognitive ability, father's occupational class and childhood BMI). Model A: adjusted for sex, birth order, cognitive ability and father's occupational class. Model B: as for model A plus adjustment for BMI at age 11.

Table S2B Birth weight and participation in leisure-time physical activity (LTPA) between 36–68 years

	OR (95% CI) of LTPA (at least once per month) across adulthood versus no LTPA	
	Model A	Model B
Birth weight group (kg)		
≤ 2.50 (n=120)	1.00 (reference)	1.00 (reference)
2.51-3.00 (n=455)	1.39 (0.96 – 2.02)	1.37 (0.95 – 1.98)
3.01-3.50 (n=974)	1.68 (1.19 – 2.39)	1.70 (1.20 – 2.41)
3.51-4.00 (n=906)	1.42 (1.00 – 2.03)	1.47 (1.03 – 2.08)
> 4.00 (n=282)	1.50 (1.00 – 2.23)	1.54 (1.04 – 2.30)

N=2737 (sample comprises those with at least one measure of LTPA and data on birth weight, ability in school sports, birth order, cognitive ability, father's occupational class and adulthood BMI). Model A: adjusted for sex, birth order, cognitive ability, father's occupational class, ability in school sports and physical health in adulthood. Model B: as for model A plus adjustment for BMI at age 36.
