# Supplementary material for: Birth Weight, School Sports Ability, and Adulthood Leisure-Time Physical Activity
Source: Med Sci Sports Exerc. Author manuscript; Available in PMC 2017 Jan 3. (PMC5207304; doi:10.1249/MSS.0000000000001077)
Supplement: Supplementary Digital Content 3 [file NIHMS69595-supplement-Supplementary_Digital_Content_3.pdf]

Supplementary Digital Content 3 Associations between birth weight and leisure-time physical activity (LTPA) at each adult age in study participants with no missing data

	OR (95% CI) of LTPA (at least once per month) versus no LTPA		
	Model 1	Model 2	Model 3
Birth weight (kg)			
LTPA age 36 years			
≤ 2.50	1.00 (reference)	1.00 (reference)	1.00 (reference)
2.51-3.00	1.71 (0.98 – 2.98)	1.58 (0.89 – 2.79)	1.55 (0.88 – 2.75)
3.01-3.50	2.16 (1.28 – 3.65)	2.00 (1.17 – 3.41)	1.95 (1.14 – 3.33)
3.51-4.00	1.78 (1.05 – 3.01)	1.56 (0.91 – 2.69)	1.54 (0.90 – 2.66)
> 4.00	1.82 (0.996 – 3.34)	1.75 (0.94 – 3.27)	1.71 (0.91 – 3.20)
LTPA age 43 years			
≤ 2.50	1.00 (reference)	1.00 (reference)	1.00 (reference)
2.51-3.00	1.34 (0.76 – 2.37)	1.25 (0.70 – 2.25)	1.23 (0.68 – 2.20)
3.01-3.50	1.96 (1.15 – 3.34)	1.86 (1.08 – 3.22)	1.80 (1.04 – 3.12)
3.51-4.00	1.77 (1.03 – 3.03)	1.63 (0.93 – 2.83)	1.60 (0.92 – 2.80)
> 4.00	1.70 (0.93 – 3.13)	1.83 (0.97 – 3.44)	1.77 (0.94 – 3.33)
LTPA age 53 years			
≤ 2.50	1.00 (reference)	1.00 (reference)	1.00 (reference)
2.51-3.00	1.65 (0.94 – 2.88)	1.48 (0.84 – 2.63)	1.47 (0.83 – 2.62)
3.01-3.50	1.96 (1.16 – 3.31)	1.77 (1.03 – 3.03)	1.73 (1.01 – 2.97)
3.51-4.00	1.96 (1.15 – 3.32)	1.69 (0.98 – 2.91)	1.68 (0.97 – 2.90)
> 4.00	1.45 (0.80 – 2.64)	1.41 (0.76 – 2.63)	1.37 (0.73 – 2.56)
LTPA age 60-64 years			
≤ 2.50	1.00 (reference)	1.00 (reference)	1.00 (reference)
2.51-3.00	2.13 (1.11 – 4.08)	1.94 (0.999 – 3.76)	1.93 (0.99 – 3.77)
3.01-3.50	2.36 (1.27 – 4.38)	2.16 (1.15 – 4.05)	2.12 (1.12 – 4.01)
3.51-4.00	2.43 (1.30 – 4.53)	2.12 (1.12 – 4.01)	2.14 (1.13 – 4.08)
> 4.00	1.94 (0.97 – 3.88)	1.86 (0.91 – 3.79)	1.83 (0.89 – 3.75)
LTPA age 68 years			
≤ 2.50	1.00 (reference)	1.00 (reference)	1.00 (reference)
2.51-3.00	1.61 (0.90 – 2.88)	1.42 (0.78 – 2.58)	1.41 (0.78 – 2.57)
3.01-3.50	1.45 (0.84 – 2.51)	1.26 (0.72 – 2.21)	1.24 (0.71 – 2.18)
3.51-4.00	1.66 (0.96 – 2.88)	1.35 (0.77 – 2.39)	1.36 (0.77 – 2.40)
> 4.00	1.34 (0.72 – 2.51)	1.15 (0.60 – 2.19)	1.13 (0.59 – 2.17)

N=1413 (sample restricted to those with complete data on leisure-time physical activity from each age across adulthood). ≤ 2.50 kg (n=65, range=1.36 to 2.50 kg; mean=2.29 kg), 2.51-3.00 kg (n=228), 3.01-3.50 kg (n=522), 3.51-4.00 kg (n=458), > 4.0 kg (n=140, range=4.09 to 5.00 kg; mean=4.31 kg). OR: Odds Ratio. 95% CI: 95% Confidence Intervals. Model 1: adjusted for sex. Model 2: adjusted for sex, birth order, father's occupational class and cognitive ability. Model 3: as for model 2 plus additional adjustment for ability in school sports and physical health in adulthood. Models at age 60-64 were also adjusted for age.
